# Early detection of pediatric bipolar disorders: a systematic review and meta-analysis

**DOI:** 10.1192/j.eurpsy.2025.1040

**Published:** 2025-08-26

**Authors:** C. Alcaino

**Affiliations:** 1 University of Edinburgh, Edinburgh, United Kingdom

## Abstract

**Introduction:**

Bipolar disorders (BD) are among the most impairing of pediatric psychiatric disorders. Even though BD symptoms may begin in adolescence, they are frequently not diagnosed until adulthood. BD screening tests could aid diagnostic assessment in paediatric populations and are supported by The International Society for Paediatric Bipolar Disorders Task Force and empirical evidence.

**Objectives:**

This review synthesizes the evidence on the accuracy of BD symptom screening tests in distinguishing bipolar disorders from other psychiatric conditions or healthy cases in pediatric populations. Additionally, it examines a wide range of potential moderators that may influence diagnostic accuracy.

**Methods:**

A systematic search was conducted across three databases (1980–2022), supplemented by searches of grey literature, citation chaining, and author contact. Data from relevant studies were combined using meta-analysis. A multilevel model was used to account for nested effect sizes, with 31 potential moderators tested in both univariate and multivariate models.

**Results:**

2,281 records were identified; 1712 titles-and-abstracts records were screened; 114 records were full-text screened; and 28 studies were included. The meta-analysis was based on all *s*=28 studies, 40 independent samples, *k*=115 effect sizes, and *n*=11,464 participants. Meta-analytic results showed that BD symptom index tests have high diagnostic accuracy in pediatric populations (g = 1.300; 95% CI: 0.982 - 1.619; p < .001) (see Fig. 1). Accuracy varied based on the comparison group, test content, test informant, and the specific scale or subscale used.

**Image:**

**Figure:**
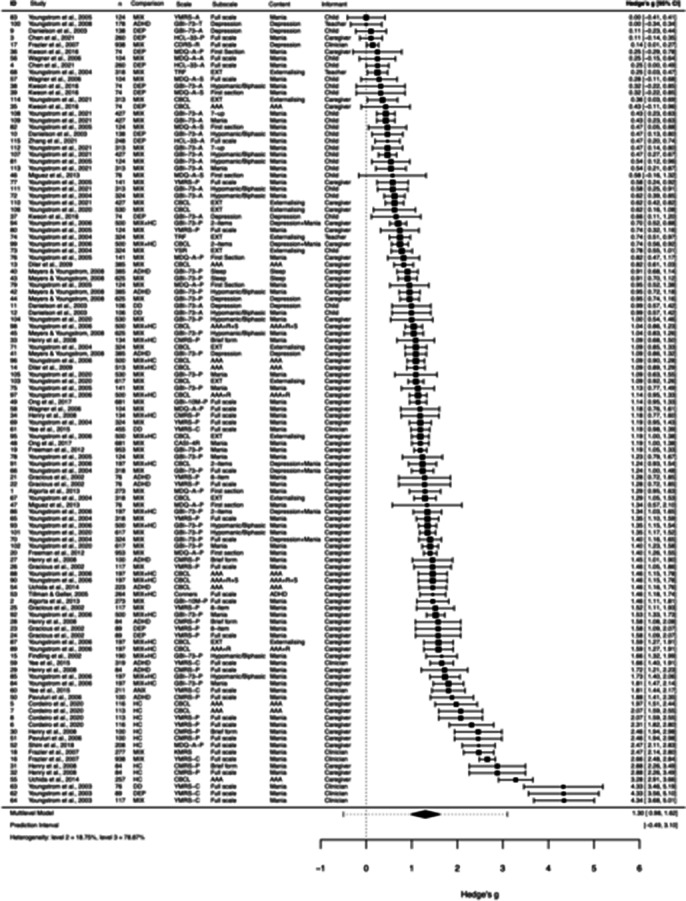

**Conclusions:**

Screening tests focusing on mania-related symptoms, caregiver reports, and psychiatric comparison groups demonstrate clinical value in identifying pediatric BD. Also, other informants and symptom content combinations may not reliably identify pediatric BD. Importantly, tests derived from studies using psychiatric comparison groups, represent BD symptom non-specificity and BD symptom overlap with other disorders (eg. ADHD and depression), providing external validity and clinical utility. Screening tests with high accuracy and clinically useful are the GBI-73-P, MDQ-A-P and the YMRS-P.

**Disclosure of Interest:**

None Declared

